# Interfacial Strengthening of Graphene/Aluminum Composites through Point Defects: A First-Principles Study

**DOI:** 10.3390/nano11030738

**Published:** 2021-03-15

**Authors:** Xin Zhang, Shaoqing Wang

**Affiliations:** 1 Shenyang National Laboratory for Materials Science, Institute of Metal Research, Chinese Academy of Sciences, Shenyang 110016, China; xzhang17b@imr.ac.cn; 2 School of Materials Science and Engineering, University of Science and Technology of China, Shenyang 110016, China

**Keywords:** Stone–Wales defect, single vacancy, double vacancy, interfacial bonding strength, mechanical properties, graphene/Al composites

## Abstract

The relationship between point defects and mechanical properties has not been fully understood yet from a theoretical perspective. This study systematically investigated how the Stone–Wales (SW) defect, the single vacancy (SV), and the double vacancy (DV) affect the mechanical properties of graphene/aluminum composites. The interfacial bonding energies containing the SW and DV defects were about twice that of the pristine graphene. Surprisingly, the interfacial bonding energy of the composites with single vacancy was almost four times that of without defect in graphene. These results indicate that point defects enhance the interfacial bonding strength significantly and thus improve the mechanical properties of graphene/aluminum composites, especially the SV defect. The differential charge density elucidates that the formation of strong Al–C covalent bonds at the defects is the most fundamental reason for improving the mechanical properties of graphene/aluminum composites. The theoretical research results show the defective graphene as the reinforcing phase is more promising to be used in the metal matrix composites, which will provide a novel design guideline for graphene reinforced metal matrix composites. Furthermore, the sp^3^-hybridized C dangling bonds increase the chemical activity of the SV graphene, making it possible for the SV graphene/aluminum composites to be used in the catalysis field.

## 1. Introduction

Metal matrix composites (MMCs), which comprise of the metal matrix and the reinforcing phase, have been in the spotlight since the early 1990s [1]. MMCs with distinctive properties can be obtained by incorporating appropriate reinforcing phase. As a vital component of MMCs, the reinforcing phase should meet the following conditions: good mechanical and functional properties as well as fine chemical stability and affinity with the metal matrix. Traditional reinforcing phases are mainly composed of carbides [2,3], metal oxides [4,5], nitrides [6,7], borides [8,9], and so on. In recent years, novel kinds of reinforcing phases, such as fullerene, carbon nanotubes, and graphene, have attracted widespread attention.

Graphene, a typical two-dimensional single-atomic-layer material consisting of sp^2^-hybridized carbon atoms [10], has attracted tremendous attention and research interest since 2004 due to its outstanding mechanical properties [11], high room temperature charge carrier mobility [12], excellent thermal conductivity [13], and theoretically large surface area [14], especially its inherent fracture strength of 130 GPa and Young’s modulus of 1 TPa. As a result, graphene is widely considered as the strongest reinforcing phase in MMCs. Recently, graphene-reinforced metal matrix composites (GMMCs) have been studied by considerable research efforts and have been shown to exhibit pronounced potential as the next generation functional and structural materials [15,16,17,18]. To date, graphene as the reinforcing phase has been added into a series of metal matrices, such as Al [19,20], Cu [21,22], Ni [23,24], Co [25], Ti [26], Mg [27], Ag [28], Fe [29], W [30], V [31], Al alloys [32,33], Mg alloys [34], Sn alloy [35], Ni–Al alloy [36], Ti–Al alloy [37], and W–Cu alloy [38]. Nevertheless, many challenges still need to be overcome in the practical applications of graphene/metal composites. It is difficult for graphene sheets to disperse uniformly into a metal matrix due to severe agglomeration caused by the strong interlayer Van der Waals interaction [39]. Moreover, it is difficult to obtain a strong interfacial bonding due to low wettability of graphene sheets with the metal matrix [40]. The final challenge is that high temperatures or very harsh processing conditions can easily destroy the structural integrity of graphene during the preparation process of graphene/metal composites [41]. Thus, how to effectively disperse graphene into a metal matrix, how to improve the graphene/metal interfacial bonding, and how to preserve the structural integrity of graphene are of great significance to the final properties of graphene/metal composites. In order to achieve the above goals, researchers have developed a variety of processing techniques for graphene/metal composites, including mechanical alloying [42], semi-powder metallurgy [43], molecular-level mixing [44], electrochemical deposition [45], in situ growth [46], and other novel techniques [47,48,49,50]. However, there exist a certain number of defects and disorders in the crystalline materials according to the second law of thermodynamics. Point defects and line defects generated during the synthesis process are the most important lattice imperfections for graphene.

Al is the most widely studied metal matrix in GMMCs, owing to its physical and mechanical properties, such as light weight, high corrosion resistance, as well as excellent electrical and thermal conductivity. Nevertheless, the interfacial bonding strength between graphene and the Al matrix is an important factor that affects the mechanical properties of composites. Due to the poor wettability of graphene with the metal matrix, graphene is difficult to form a strong bond with the metal matrix, thus resulting in the poor performance of composite materials [40]. A few recent experimental and theoretical works demonstrate that the defects in graphene enhance the mechanical properties of graphene/metal composites significantly [51,52,53,54]. However, to our knowledge, the interfacial strengthening mechanism between the graphene with the point defects and the Al matrix has not been fully understood yet from a theoretical insight.

In this work, the way in which the three typical point defects (Stone–Wales (SW), single vacancy (SV), and double vacancy (DV)) affect the mechanical properties of graphene/Al composites was studied systematically through the first-principles calculations. Different from the pristine graphene, more sophisticated adsorption sites need to be considered due to the existence of the defects in graphene, indicating that we need to construct a series of different interfacial configurations. Consequently, the interfacial bonding energy, the equilibrium interlayer distance, the minimum interatomic distance, and the buckling of these different interfacial configurations were investigated systematically through the first-principles calculations to better understand how the point defects affect the mechanical properties of graphene/Al composites. Furthermore, the differential charge density was also calculated to give an intuitive illustration of the interfacial electronic structure and charge transfer to understand the interfacial strengthening mechanism between graphene and the Al matrix.

## 2. Computational Methods

**Figure 1:**
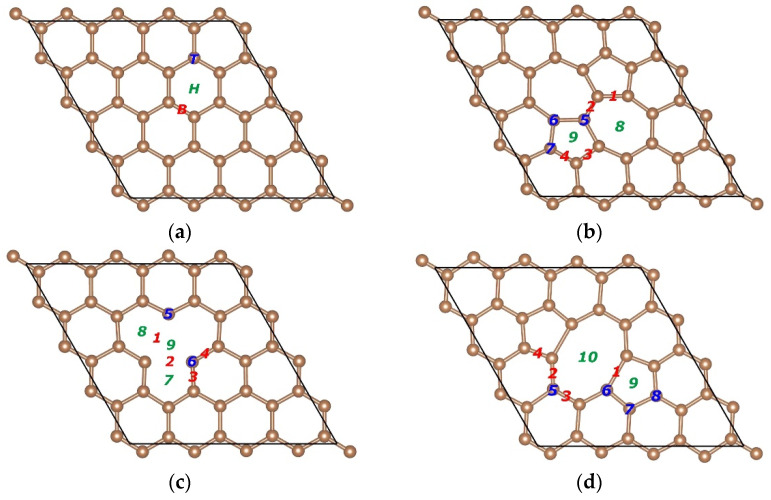
Structure of the pristine graphene, the Stone–Wales (SW) defect graphene, the single vacancy (SV) graphene, and the double vacancy (DV) graphene prepared for density function theory (DFT) calculations and adsorption sites on graphene. (**a**) Bridge (red), Top (blue), and Hollow (green) on the pristine graphene. (**b**) Bridge 1–4, Top 5–7, and Hollow 8–9 on the SW defect graphene. (**c**) Bridge 1–4, Top 5–6, and Hollow 7–9 on the SV graphene. (**d**) Bridge 1–4, Top 5–8, and Hollow 9–10 on the DV graphene.

Pristine graphene is composed of hexagonal rings, which serve as the building blocks for the sp^2^-bonded low-dimensional carbon structures, as shown in Figure 1a. Two π-bonded carbon atoms in the pristine graphene are rotated by 90° and thus form 2 pentagons and 2 heptagons, namely, the SW defect, as shown in Figure 1b. It can be found that the reconstructed graphene reserves the original number of atoms and does not contain any dangling bonds. If one carbon atom is missing, there will be an SV defect in graphene, as shown in Figure 1c. If 2 carbon atoms are missing, there will be a DV defect in graphene, as shown in Figure 1d. It can be observed that dangling bonds will remain in the SV defect, owing to the geometrical reasons. However, the dangling bonds can be healed in the DV defect due to the connectivity of carbon atoms. In general, there are 3 typical adsorption sites considered when a metal atom adsorbs on the pristine graphene. The 3 typical adsorption sites are on the top of a carbon atom (Top, T), at the center of a hexagon ring (Hollow, H), and in the middle of a carbon–carbon covalent bond (Bridge, B), as shown in Figure 1a. Different from the pristine graphene, more sophisticated adsorption sites need to be considered due to the existence of the defects in graphene, as shown in Figure 1b–d. Before constructing the interfacial configurations, we first performed the structural optimization for graphene with 3 kinds of point defects and obtained the formation energy of the specific defects. The formation energies of the Stone–Wales defect, the SV vacancy, and the DV vacancy were 5.39, 7.48, and 7.93 eV, respectively, which are in good agreement with the results reported in the literature [55,56,57,58]. The results show that the Stone–Wales defect is the easiest to form and the double vacancy is the most difficult to form.

The excessive lattice constant mismatch of graphene adsorbed on metal surfaces will lead to the initial stress in the interior of graphene sheet. As a result, it is impossible to determine whether the buckling of graphene after adsorption on metal surfaces is due to the interactions between graphene and the metal surface, or due to the release of the initial stress in the interior of graphene sheet. Therefore, the lattice constant mismatch is a priority factor in constructing the interfacial configuration. A metal surface was constructed using a slab that was composed of a finite number of layers of metal and a vacuum region in the direction perpendicular to the layers. We constructed the graphene/metal interface supercell from a slab consisting of six layers of metal atoms, with monolayer graphene adsorbed from the top of the slab. The vacuum region was 15 Å. It should be noted that the upper layer film is usually thinner than the metal substrate and the film tends to match the lattice constant of the metal substrate in the experiment [59,60], and thus the lattice constant of metal unit cell was fixed to construct the interface supercell. The 4 × 4 pristine graphene unit cell and 4 × 4 defective graphene (SW defect, SV, and DV) unit cells were adjusted to the 4 × 4 unit cell of the Al (111) surface by the Materials Studio Software. The approximation made by the above matching procedure was reasonable because the lattice mismatch of graphene was only −0.251%.

Density function theory (DFT) calculations were carried out by the Vienna ab initio simulation package (VASP) [61]. We selected the local density approximation (LDA) in order to describe the exchange correlation effect because LDA can exhibit better performance in predicting the bonding behavior between carbon nanostructures and metals than the generalized gradient approximation (GGA) [62,63]. The projector-augmented wave (PAW) method was selected to describe the electron–ion interactions [64,65]. The spin polarization was also taken into account in the calculation. A dipole correction was applied in order to avoid spurious interactions between periodic images of the slab [66]. Graphene mainly interacts with the 2 topmost layers of metal atoms. Thus, we permitted the positions of the top 2 layers of metal atoms as well as those of carbon atoms to relax freely but fixed the positions of the bottom 4 layers of metal atoms to simulate the metal bulk in optimizing the configuration. The criteria used in our geometrical optimization are as follows. The maximum residual force on every atom was no more than 0.01 eV/Å with respect to ionic relaxation and the total energy of the system was converged to within 1.0 × 10^−5^ eV.

## 3. Results and Discussion

### 3.1. Graphene-Al (111) Bonding

The detailed parameters of the pristine graphene/Al (111) and the defective graphene/Al (111) interfaces are listed in Table 1, Table 2, Table 3 and Table 4. The interfacial bonding energy $E_{b}\;$is an important criterion to analyze the interfacial bonding strength between graphene and metal matrix. The interfacial bonding energy $E_{b}$ in this study is calculated as follows:

$$E_{b}=E_{C}+E_{M}-E_{C-M}$$

where $E_{C}$, $E_{M}$, and $E_{C-M}$ are the total energy of the pristine graphene or the defective graphene, the bare slab, and the adsorption system, respectively. A positive $E_{b}$ illustrates that the adsorption system should be stable according to the definition. Moreover, a larger $E_{b}$ demonstrates the adsorption system is more stable. The equilibrium interlayer distance $d_{C-\mathrm{Al}}$ is defined as the average distance from the topmost layer of Al to graphene in the vertical direction of the interface. The minimum interatomic distance $d_{\min}$ is defined as the minimum atomic distance from the topmost layer atoms of Al to the carbon atoms. The buckling $B_{C}$ and $B_{\mathrm{Al}}$ are defined as the maximum height difference of the graphene sheet and the topmost layer of Al in the vertical direction of the interface after optimization, respectively.

For the pristine graphene, $E_{b}$ and $d_{C-\mathrm{Al}}$ obtained from configurations B, and T and H are essentially the same according to Table 1, which is on the whole in agreement with the results in the literature [67]. In addition, there was no distinct buckling of the graphene sheet found from the two configurations. A slight difference is that $d_{\min}$ obtained from the second configuration was slightly smaller than that from the first configuration, but $B_{\mathrm{Al}}$ obtained from the second configuration was larger than that from the first configuration, indicating that the interfacial bonding strength is stronger when the Al atoms lie on the top of the carbon atoms compared to other adsorption sites.

For the SW defect graphene, we considered more sophisticated adsorption sites due to the existence of two pentagons and two heptagons, as shown in Figure 1b. Table 2 gives the detail parameters of different starting configurations. The increase of $E_{b}$ and the decrease of $d_{C-\mathrm{Al}}$ show that the SW defect can effectively enhance the interfacial bonding strength between graphene and the Al matrix. Furthermore, the obvious increase in $B_{C}$ and $B_{\mathrm{Al}}$ also proves that the interfacial bonding strength was improved significantly. Compared with the pristine graphene, the significant change of $d_{\min}$ indicates that the carbon atoms may have formed covalent bonds with the Al atoms. Compared with other configurations, $E_{b}$, $B_{C}$, and $B_{\mathrm{Al}}$ obtained from configurations B4 and H8 were smaller, which demonstrates that the interfacial bonding strength was relatively weak. The larger $d_{C-\mathrm{Al}}$ and $d_{\min}$ can also further confirm that the interfacial bonding strength obtained from configurations B4 and H8 was not as strong as that from other configurations. Moreover, the SW defect did provide help for improving the interfacial bonding strength between graphene and the Al matrix. It is clear that $E_{b}$, $d_{C-\mathrm{Al}}$, $d_{\min}$, $B_{C}$, and $B_{\mathrm{Al}}$ obtained by optimizing from configurations B1, B2, B3, T5, T6, T7, and H9 were the same. In addition, $E_{b}$, $d_{C-\mathrm{Al}}$, $d_{\min}$, $B_{C}$, and $B_{Al}$ obtained by optimizing from configurations B4 and H8 were the same. Therefore, we believe that there were two possible equilibrium configurations after the structural optimization. The possible equilibrium configurations are given in Section 3.2.

Different from the SW defect graphene, the SV graphene has local magnetic moments, owing to the existence of dangling bonds according to the literature [68]. The spin polarization was also considered in this calculation. The calculation result shows that the SV graphene did have local magnetic moments. The key parameters at different adsorption sites are listed in Table 3. To our surprise, $E_{b}$ of the SV graphene/Al (111) interface was approximately four times that of the pristine graphene/Al (111) interface. In addition, the significant decrease in $d_{C-\mathrm{Al}}$ and $d_{\min}$ and the significant increase in $B_{C}$ and $B_{\mathrm{Al}}$ show that the SV can play a vital role in improving the interfacial bonding strength between graphene and the Al matrix. Furthermore, $E_{b}$ of the SV graphene/Al (111) interface was more than twice that of the DV graphene/Al (111) interface. These results indicate that the SV was more effective to enhance the interfacial bonding strength between graphene and the Al matrix. It is worth noting that the local magnetic moment in the SV graphene disappeared after adsorption, demonstrating that the dangling bonds no longer existed. In other words, the carbon atoms may form covalent bonds with the Al atoms. Notably, $E_{b}$, $d_{C-\mathrm{Al}}$, $d_{\min}$, $B_{C}$, and $B_{\mathrm{Al}}$ obtained by optimizing from configurations B1, B2, B3, B4, T6 and H8, and H9 were the same, except for the configuration T5 and H7, indicating that there may only be two equilibrium configurations after the structural optimization. We provide the possible equilibrium configurations in Section 3.2.

The DV is formed in the graphene sheet when two carbon atoms are missed. Different from the SV, the dangling bonds can be healed for the connectivity of carbon atoms. Therefore, no dangling bonds remain in the DV graphene, indicating that the chemical activity of the DV graphene is not good as that of the SV graphene. It can be seen from Table 4 that the larger$\;E_{b}$, $B_{C}$, and $B_{\mathrm{Al}}$ as well as the smaller $d_{C-\mathrm{Al}}$ and $d_{\min}$ elucidate that the interfacial bonding strength between the DV graphene and the Al matrix was stronger than that between the pristine graphene and the Al matrix. According to the data in Table 4, we find that the interfacial bonding strength obtained from configurations B2, B3, T6, and T7 ranked first and the interfacial bonding strength obtained from the configuration H10 ranked last. Combined with the data in Table 4, we infer that there are five possible equilibrium configurations after the structural optimization. This is further discussed in Section 3.2.

### 3.2. Configurations Analysis

**Figure 2:**
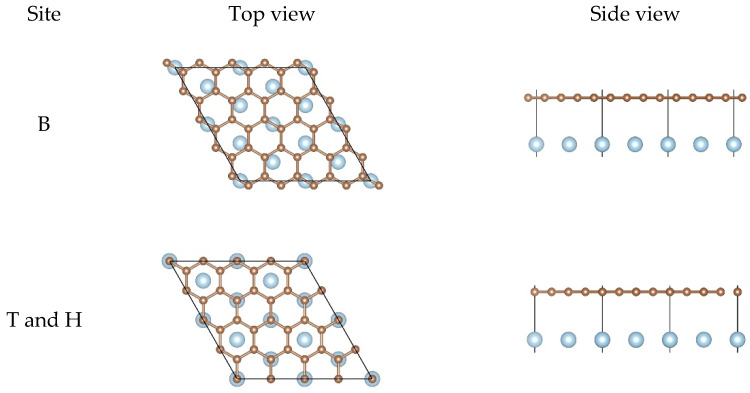
Top views and side views of the pristine graphene on the Al (111) surface after the structural optimization.

**Figure 3:**
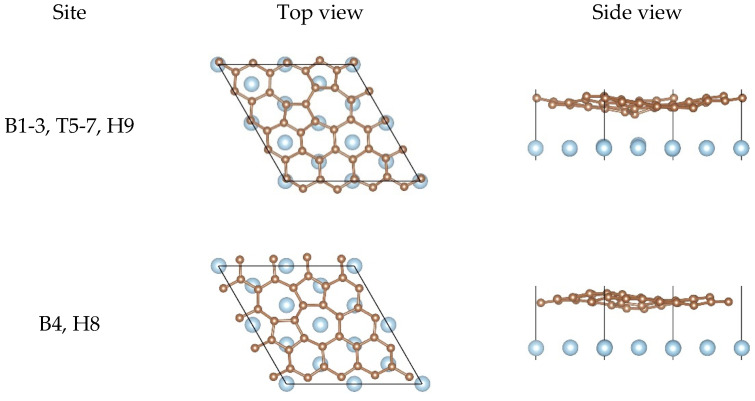
Top views and side views of the SW defect graphene on the Al (111) surface after the structural optimization.

**Figure 4:**
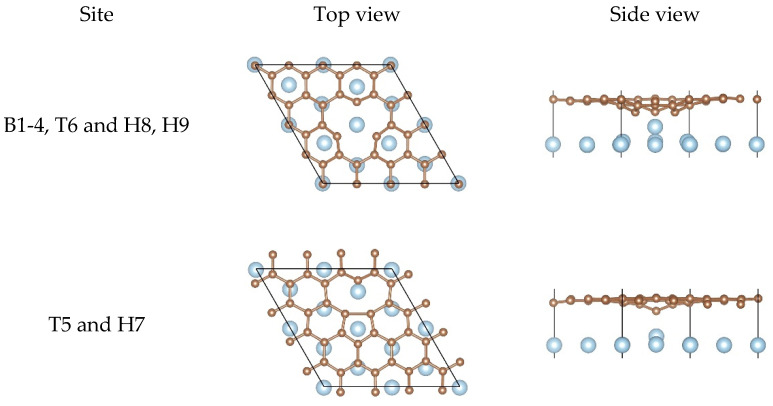
Top views and side views of the SV graphene on the Al (111) surface after the structural optimization.

**Figure 5:**
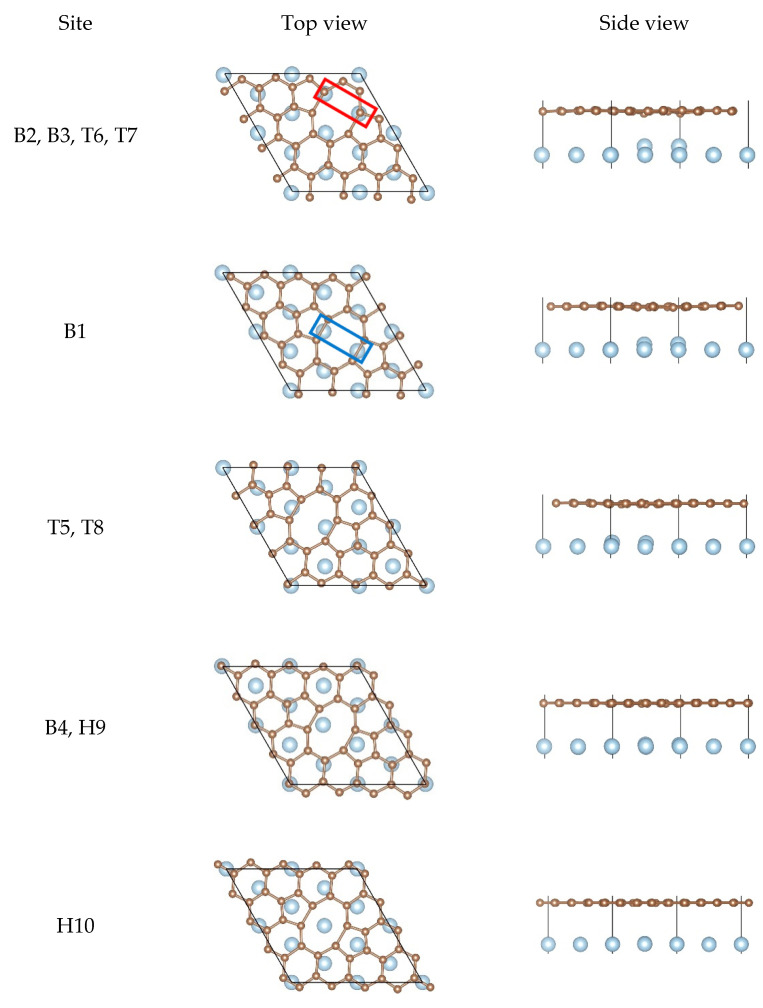
Top views and side views of the DV graphene on the Al (111) surface after the structural optimization.

According to the different adsorption sites shown in Figure 1, we constructed a series of different corresponding adsorption configurations in order to study the interfacial bonding strength between graphene and the Al matrix. In detail, all the adsorption configurations were constructed from a slab consisting of six layers of Al atoms, with monolayer graphene adsorbed from the top side of the slab. After the structural optimization, we found that the equilibrium configurations were only few in number. Therefore, we only discuss the equilibrium configurations of the pristine graphene, the SW defect graphene, the SV graphene, and the DV graphene adsorbed on the Al (111) surface, which are shown in Figure 2, Figure 3, Figure 4 and Figure 5, respectively.

For the pristine graphene/Al (111) interface in Figure 2, there was hardly any change in the relative positions between the carbon atoms and the Al atoms from the top views and there was almost no buckling of the graphene sheet and the topmost layer of Al from the side views. However, the equilibrium interlayer distance $d_{C-\mathrm{Al}}$ increased significantly compared to the initial interfacial distance. These results show that the interfacial bonding strength between the pristine graphene and the Al (111) surface was weak.

When it comes to the SW defect graphene/Al (111) interface, there are two kinds of equilibrium configurations found, as shown in Figure 3. For the first equilibrium configuration, we found that most Al atoms lay below carbon atoms and other Al atoms were located at the center of hexagon rings. In addition, we could observe that there was distinct buckling of the graphene sheet and the topmost layer of Al from the side views, especially where the carbon atoms were in contact with the Al atoms at the defect. However, for the second equilibrium configuration, it was clearly found that there were two Al atoms located at the center of two heptagon rings. Moreover, $B_{C}$ was obvious but $B_{\mathrm{Al}}$ was very small, which indicates that the interfacial bonding strength of the configuration was not as strong as that of the first configuration. In other words, the first configuration was more stable. In any case, the SW defect did improve the interfacial bonding strength between graphene and the Al matrix, which is also consistent with our calculation results in Table 2.

For the SV graphene/Al (111) interface, two non-equivalent equilibrium configurations could be observed, as shown in Figure 4. For the first configuration, there was always one Al atom lying just below the carbon vacancy and other Al atoms lying either below the carbon atoms or at the center of the hexagon rings. Notably, $B_{\mathrm{Al}}$ was particularly remarkable and the Al atom had been pulled out from the original position completely, which was enough to confirm how strongly the SV graphene interacted with the Al matrix. As for the second configuration, the SV graphene should undergo the reconstruction of graphene lattice after adsorption due to Jahn–Teller distortion according to the literature [69]. By contrast, half of the Al atom was pulled out from the original position in the second configuration while the whole Al atom was completely pulled out from the original position in the first one, indicating the interfacial bonding strength of the first configuration was much stronger than the second configuration.

In the case of the DV graphene/Al (111) interface, there were five possible equilibrium configurations found, as shown in Figure 5. The different buckling of the graphene sheet and the topmost layer of Al were found from the side views. As for the topmost layer of Al, different degree of buckling was found in the first three configurations, while there was almost no buckling found in the last two configurations, which indicates that the interfacial bonding strength of the first three configurations was much stronger than that of the last two configurations. For the first configuration, there were always two Al atoms found in the nearest neighbor of two carbon atoms at the defect, as shown in the red line frame in Figure 5, and other Al atoms lay below the carbon atoms as much as possible. For the second configuration, there were two Al atoms found in the middle of carbon–carbon covalent bonds at the defect, as shown in the blue line frame in Figure 5. It seemed to be the same for the third configuration and the fourth configuration from the top view, but different buckling in the topmost layer of Al was seen from the side views, which is consistent with the different interfacial bonding energies of the two configurations. For the last configuration, there was an Al atom found in the center of the octagon ring, and two Al atoms lying below the pentagon rings. 

In summary, we found that the existence of defects in graphene did enhance the interfacial bonding strength significantly and thus improved the mechanical properties of graphene/Al composites, especially the SV graphene. The above results, such as $d_{C-\mathrm{Al}}$, $B_{C}$, and $B_{\mathrm{Al}}$, made us believe that the carbon atoms at the defect may form the covalent bonds with the Al atoms. The differential charge density was the most powerful evidence to prove whether the carbon atoms form the covalent bonds with the Al atoms or not. Therefore, we discuss the differential charge density in the following paragraphs.

### 3.3. Differential Charge Density Analysis

To understand the interfacial strengthening mechanism between graphene and the Al matrix, we also calculated the differential charge density in order to observe the interfacial electronic structure and charge transfer more intuitively. The differential charge density in this work was calculated as follows:

$$\Delta \rho =\rho_{C-M}-\rho_{C}-\rho_{M}$$

where $\rho_{C-M}$, $\rho_{C}$, and $\rho_{M}$ are the electronic charge density of the adsorption system, the isolated defective graphene, and the clean surface of Al, respectively. In the calculations of the latter two quantities, the atomic positions were kept fixed at precisely the same positions as they were in the adsorption system. As is known, a significant sign of forming a covalent bond is numerous charge accumulation at the center of two adjacent atoms. On the contrary, no covalent bond was formed.

**Figure 6:**
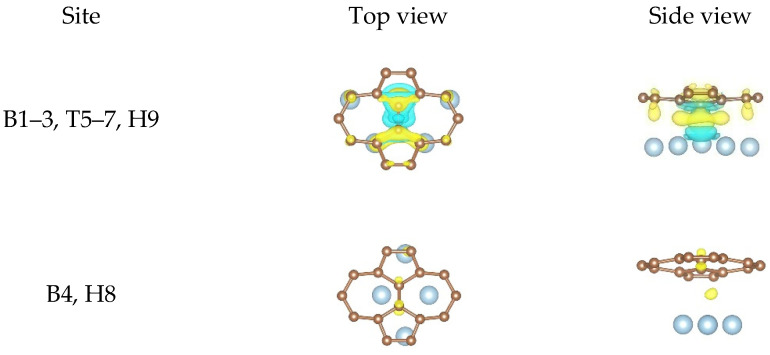
The local differential charge density plots at the SW defect graphene/Al (111) interface. The yellow/blue colors mark an increase/decrease of the charge density, respectively. Iso surfaces correspond to 3 × 10^−3^ e/Å^3^.

**Figure 7:**
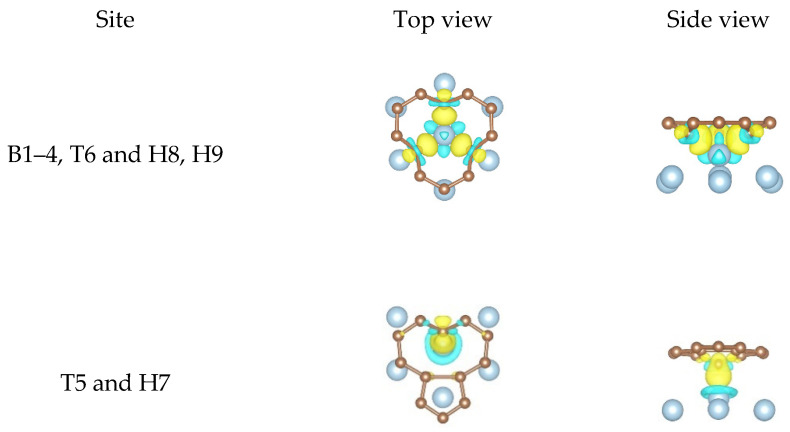
The local differential charge density plots at the SV graphene/Al (111) interface. The yellow/blue colors mark an increase/decrease of the charge density, respectively. Iso surfaces correspond to 6 × 10^−3^ e/Å^3^.

**Figure 8:**
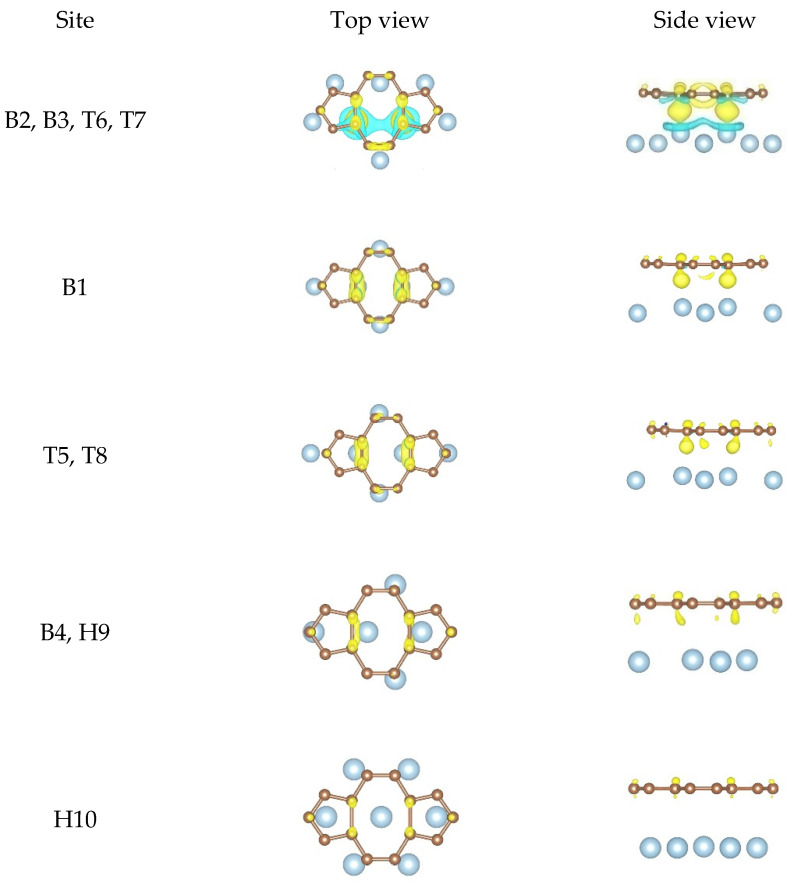
The local differential charge density plots at the DV graphene/Al (111) interface. The yellow/blue colors mark an increase/decrease of the charge density, respectively. Iso surfaces correspond to 3 × 10^−3^ e/Å^3^.

No charge redistribution around the atoms and no charge accumulation at the center of the Al atoms and the C atoms could be observed according to the differential charge density results at the pristine graphene/Al (111) interface. In other words, the interfacial bonding between the pristine graphene and Al (111) surface was only Van der Waals force. Therefore, we did not give the differential charge density plots induced by the adsorption of the pristine graphene on the Al (111) surface here. Unlike the pristine graphene, obvious charge transfer was observed between the defective graphene and the Al (111) surface and occurred mainly between the carbon atoms and the Al atoms at the defect. In order to observe the charge redistribution and the charge accumulation more intuitively, we only gave the local differential charge density plots of the non-equivalent equilibrium configurations between the carbon atoms and the Al atoms at the defect, as shown in Figure 6, Figure 7 and Figure 8.

In the case of the SW defect graphene/Al (111) interface, almost no charge redistribution and charge accumulation was observed from the second configuration, while clear charge redistribution and large amounts of charge accumulation was observed from the first configuration, as shown in Figure 6. This shows that the interfacial bonding strength obtained from the second configuration was weaker than that of the first configuration, which was also consistent with the bonding energy results in Table 2. It was seen clearly from the first configuration that the carbons atoms formed the covalent bonds with the Al atoms due to extensive charge accumulation at the center of the carbon atoms and the Al atoms, which proved that the interfacial bonding strength between the SW defect graphene and the Al (111) surface was stronger than that between the pristine graphene and the Al (111) surface.

When it comes to the SV graphene/Al (111) interface, we give the local differential charge density plots of the two non-equivalent equilibrium configurations as shown in Figure 7. For the first configuration, a great deal of charge accumulation between an Al atom and three carbon atoms was observed, illustrating that the three carbon atoms formed three covalent bonds with the Al atom. As is known, Al atom (3s^2^ 3p^1^) has only one unpaired electron, and thus it is impossible for the Al atom to form three covalent bonds with three carbon atoms directly. Therefore, we infer that the s orbital and two p orbitals of the Al atom hybridized with each other in order to form three identical sp^2^-hybridized orbitals, which is similar to the phenomenon that three carbon atoms can form three covalent bonds with one sd^2^-hybridized Ti atom reported in the previous work of our group [70]. The optimized geometric configuration shows that the three bond lengths between three carbon atoms and the Al atom at the defect were 1.943, 1.946, and 1.946 Å, indicating that the three covalent bonds were completely identical. However, the three carbon atoms and the Al atom were not in the same plane, that is, the spatial configuration of the sp^2^-hybridized Al atom was not a plane equilateral triangle, which was different from the well-known sp^2^ hybridization. We think the reason for this phenomenon was that too large Al atomic radius (than carbon atom) and too small vacancy space made the Al atom unable to move exactly to the carbon vacancy. As for the three carbon atoms that formed covalent bonds with the Al atom, they formed two covalent bonds with two carbon atoms before the structural optimization. In addition to the charge accumulation at the center of the three carbons and the Al atom after the structural optimization, the charge density in the other directions of the three carbon atoms was also clearly found from the side view, indicating the formation of the C dangling bonds. These results indicate the hybridization state of the three carbon atoms changed from the original sp^2^ hybridization to the current sp^3^ hybridization. The existence of the sp^3^-hybridized C dangling bonds was able to increase the chemical activity of the SV graphene, thus making it possible for the SV graphene/Al composite to be used in the catalysis field. As for the second configuration, we found that only a carbon atom formed a covalent bond with an Al atom, showing that the interfacial bonding strength obtained from the second configuration was weaker than that from the first one. These results also agree with our calculation results in Table 3.

For the DV graphene/Al (111) interface, the local differential charge density plots of the equilibrium configurations can be divided into the following cases, as shown in Figure 8. There were large amounts of charge accumulation found between carbon atoms and Al atoms according to the first configuration. In the second configuration, there was some charge accumulation observed. These results show that the carbon atoms formed covalent bonds with the Al atoms. However, there was little charge accumulation found from the other three configurations. It can be concluded that the interfacial bonding strength obtained from the first configuration was strongest while the interfacial bonding strength obtained from the last configuration was weakest. Moreover, the interfacial bonding strength at the DV graphene/Al (111) interface was stronger than that at the pristine graphene/Al (111) interface. In addition, we found that the charge distribution and the charge accumulation degree were different, which can also prove that the equilibrium configurations were different. Therefore, these results can verify the rationality of our calculation results in Table 4. 

Subsequently, we calculated the projected density of states to further verify the formation of covalent bonds, the sp^2^ hybridization of the Al atom, and the sp^3^ hybridization of the carbon atom at the defect. The calculated results prove the rationality of our differential charge density analysis. Here, we do not give the projected density of states plots because the detailed analysis processes have been reported in the previous work of our group [70]. Therefore, we will not go into it again.

## 4. Conclusions

We systematically investigated how the SW defect, the SV, and the DV as three typical point defects affect the mechanical properties of graphene/Al composites through the first-principles calculations. The interfacial bonding energy, the equilibrium interlayer distance, the minimum interatomic distance, and the buckling were calculated in order to further understand the relationship between the point defects and the mechanical properties. The results demonstrate that the point defects significantly increased the interfacial bonding energy and the buckling while decreasing the equilibrium interlayer distance and the minimum interatomic distance, especially the SV. The interfacial bonding energy of graphene containing the SV was approximately four times that of the pristine graphene. In other words, the point defects enhanced the interfacial bonding strength significantly and thus improved the mechanical properties of graphene/Al composites. To further understand the interfacial strengthening mechanism, we also performed the electronic structure analyses. The differential charge density shows that there was numerous charge accumulation found at the center of the carbon atoms and the Al atoms at the defects, confirming that the carbon atoms formed the strong covalent bonds with the Al atoms, which is the most fundamental reason for enhancing the mechanical properties of graphene/Al composites. It is worth noting that three covalent bonds formed between three carbon atoms and one sp^2^-hybridized Al atom were observed at the SV graphene/Al (111) interface, and the hybridization state of the three carbon atoms changed from the original sp^2^ hybridization to the current sp^3^ hybridization. These extensive calculations demonstrate that the defective graphene as the reinforcing phase is more promising in terms of use in the metal matrix, which offers a new insight to guide the design of graphene/metal composites.

## Figures and Tables

**Table 1:** The key parameters of the pristine graphene/Al (111) interface after optimization.

Type	Site	$E_{b}\left(eV\right)$	$d_{C-Al}$ (Å)	$d_{min}$ (Å)	$B_{C\;}$ (Å)	$B_{Al}$ (Å)
Pristine Graphene	B	1.154	3.442	3.506	0.0037	0.0078
	T and H	1.160	3.426	3.415	0.0038	0.0235

**Table 2:** The key parameters of the SW defect graphene/Al (111) interface after optimization.

Type	Site	$E_{b}\;\left(eV\right)$	$d_{C-Al}$ (Å)	$d_{min}$ (Å)	$B_{C\;}$ (Å)	$B_{Al}$ (Å)
SW	B1–3, T5–7, H9	2.020	3.085	2.205	1.2883	0.2523
	B4, H8	1.690	3.372	2.878	1.0828	0.0654

**Table 3:** The key parameters of the SV graphene/Al (111) interface after optimization.

Type	Site	$E_{b}\;\left(eV\right)$	$d_{C-Al}$ (Å)	$d_{min}$ (Å)	$B_{C\;}$ (Å)	$B_{Al}$ (Å)
SV	B1–4, T6 and H8, H9	4.625	2.854	1.943	1.0403	1.3278
	T5 and H7	2.890	3.127	1.987	0.9433	0.6311

**Table 4:** The key parameters of the DV graphene/Al (111) interface after optimization.

Type	Site	$E_{b}\;\left(eV\right)$	$d_{C-Al}$ (Å)	$d_{min}$ (Å)	$B_{C}$ (Å)	$B_{Al}$ (Å)
DV	B2, B3, T6, T7	1.967	3.001	2.328	0.2371	0.6121
	B1	1.934	3.012	2.745	0.1106	0.4029
	T5, T8	1.841	3.026	2.875	0.1016	0.3201
	B4, H9	1.755	3.068	3.048	0.0536	0.1710
	H10	1.691	3.143	3.199	0.0219	0.0878

## Data Availability

The data presented in this study are available on request from the corresponding author. The data are not publicly available due to the large amount of the calculation data.
